# Determinants of Intima-Media Thickness in the Young: The ALSPAC Study

**DOI:** 10.1016/j.jcmg.2019.08.026

**Published:** 2021-02

**Authors:** Scott T. Chiesa, Marietta Charakida, Georgios Georgiopoulos, Frida Dangardt, Kaitlin H. Wade, Alicja Rapala, Devina J. Bhowruth, Helen C. Nguyen, Vivek Muthurangu, Rukshana Shroff, George Davey Smith, Debbie A. Lawlor, Naveed Sattar, Nicholas J. Timpson, Alun D. Hughes, John E. Deanfield

**Affiliations:** a Vascular Physiology Unit, UCL Institute of Cardiovascular Science, London, United Kingdom; b Division of Imaging Sciences and Biomedical Engineering, King’s College London, United Kingdom; c 1st Department of Cardiology, National and Kapodistrian University of Athens, Hippokration Hospital, Athens, Greece; d Department of Paediatric Physiology, The Queen Silvia Children’s Hospital, The Sahlgrenska Academy and University Hospital, Gothenburg, Sweden; e MRC Integrative Epidemiology Unit (IEU), The University of Bristol, United Kingdom; f Population Health Sciences, Bristol Medical School, Faculty of Health Sciences, University of Bristol, Bristol, United Kingdom; g Centre for Cardiovascular Imaging, UCL Institute of Cardiovascular Science, London, United Kingdom; h Nephrology Unit, Great Ormond Street Hospital for Children NHS Foundation Trust, London, United Kingdom; i Institute of Cardiovascular and Medical Science, University of Glasgow, United Kingdom; j Department of Population Science and Experimental Medicine, UCL Institute of Cardiovascular Science, London, United Kingdom; k MRC Unit for Lifelong Health and Ageing at UCL, London, United Kingdom

**Keywords:** ALSPAC, fat-free mass, intima-media thickness, systolic blood pressure, BMI, body mass index, BP, blood pressure, CI, confidence intervals, cIMT, common carotid artery intima-media thickness, CVD, cardiovascular disease, CWS, circumferential wall stress, FFM, fat-free mass, FM, fat mass, LCGMM, latent class growth mixture modelling, UGFUS, ultra-high frequency ultrasound technology

## Abstract

**Objectives:**

This study characterized the determinants of carotid intima-media thickness (cIMT) in a large (n > 4,000) longitudinal cohort of healthy young people age 9 to 21 years.

**Background:**

Greater cIMT is commonly used in the young as a marker of subclinical atherosclerosis, but its evolution at this age is still poorly understood.

**Methods:**

Associations between cardiovascular risk factors and cIMT were investigated in both longitudinal (ages 9 to 17 years) and cross-sectional (ages 17 and 21 years) analyses, with the latter also related to other measures of carotid structure and stress. Additional use of ultra-high frequency ultrasound in the radial artery at age 21 years allowed investigation of the distinct layers (i.e., intima or media) that may underlie observed differences.

**Results:**

Fat-free mass (FFM) and systolic blood pressure were the only modifiable risk factors positively associated with cIMT (e.g., mean difference in cIMT per 1-SD increase in FFM at age 17: 0.007 mm: 95% confidence interval [CI]: 0.004 to 0.010; p < 0.001), whereas fat mass was negatively associated with cIMT (difference: −0.0032; 95% CI: 0.004 to −0.001; p = 0.001). Similar results were obtained when investigating cumulative exposure to these factors throughout adolescence. An increase in cIMT maintained circumferential wall stress in the face of increased mean arterial pressure when increases in body mass were attributable to increased FFM, but not fat mass. Risk factor−associated differences in the radial artery occurred in the media alone, and there was little evidence of a relationship between intimal thickness and any risk factor.

**Conclusions:**

Subtle changes in cIMT in the young may predominantly involve the media and represent physiological adaptations as opposed to subclinical atherosclerosis. Other vascular measures may be more appropriate for the identification of arterial disease before adulthood.

Lifetime management of cardiovascular disease (CVD) risk factors is recognized as an important strategy for reducing future population burden of disease (1). In early life, the absence of clinical endpoints necessitates the use of intermediate cardiovascular phenotypes in population studies and clinical trials to understand the initiation and progression of early arterial disease and its response to interventions. Increased common carotid intima-media thickness (cIMT) is a well-established marker for future adverse CVD outcomes in older populations and certain clinical conditions (2) and is frequently also used in young population cohorts to link emerging CVD risk factors to the earliest development of subclinical atherosclerosis (3, 4, 5, 6).

However, conventional cIMT is a composite measure of both intimal and medial layers and is therefore unable to determine whether changes in arterial wall thickness represent early atherosclerotic changes within the intimal layer, a remodeling response within the medial layer, or a combination of both. Although numerous studies have shown associations between well-established CV risk factors and cIMT (7), others have also reported associations with height, lean tissue mass, and vessel diameter (8, 9, 10), which suggests that physiological remodeling due to increased body size or growth may account for at least some of the changes commonly attributed to subclinical disease. These factors may be particularly relevant in the young, when cumulative exposure to CVD risk factors is low and comorbidities that increase atherosclerotic risk are largely absent.

Advances in ultra-high frequency ultrasound technology (UHFUS) now permit the individual layers of the arterial wall to be studied in detail (11, 12, 13, 14). In an extensively phenotyped longitudinal cohort of healthy young people recruited to the ALSPAC (Avon Longitudinal Study of Parents and Children) trial, we combined this novel UHFUS technology in the radial artery with both cross-sectional and longitudinal analyses of cIMT to characterize the determinants of arterial wall changes in adolescence and young adulthood, and related these changes to other measures of carotid artery structure and biomechanical stress to identify potential adaptive and maladaptive changes at this age.

## Methods

### Cohort description

ALSPAC is a prospective birth cohort study investigating factors that influence normal childhood development and growth. The cohort and study design have been described in detail previously (15,16), and a brief description is provided in the Supplemental Appendix. Ethical approval for all aspects of this study was obtained from the ALSPAC Ethics and Law Committee and the Local Research Ethics Committee, and conformed to the Declaration of Helsinki. If the child was younger than age 16 years, informed written consent was obtained from the parent/guardian alongside assent from the child. When age 16 years or older, participants provided their own informed written consent.

### Study design

Using a combination of both cross-sectional and longitudinal analyses from an age range spanning childhood (9 years) to young adulthood (21 years), we used 3 separate approaches to characterize determinants of IMT in a large cohort of young people free from clinical disease.

In 5,215 adolescents (mean age: 18 years) attending the ALSPAC Teen Focus 4 Clinic (ALSPAC@17), we investigated cross-sectional relationships between a range of CV risk factors (body composition, blood pressure [BP], lipids, insulin, glucose, inflammatory markers, lifestyle behaviors, and socioeconomic circumstances) and cIMT. In addition, we explored how these differences in cIMT were related to other measures of structure and stress within the carotid artery (lumen diameter, IMT/lumen ratio, and vessel circumferential wall stress).

Based on findings from the cross-sectional analysis, we next used repeat measures of body composition and BP in 4,561 participants (collected on 2 to 5 occasions between the ages of 9 and 17 years) to determine the effect of their cumulative exposure throughout adolescence on cIMT at 17 years.

Finally, further detailed phenotyping in 436 participants recalled at age 21 years allowed us to: 1) assess whether increased duration of exposure to risk factors in young adulthood altered relationships seen in adolescence; and 2) use UHFUS in a subsample of these participants to investigate the contribution of intimal and medial layers to arterial changes. Details of this cohort have been described previously (17), and a comparison of baseline characteristics between those who attended recall at age 21 years versus those who did not is shown in Supplemental Table 1.

### Arterial phenotypes

#### Carotid artery structure and stress using high-frequency ultrasound

Common carotid artery lumen diameter, cIMT, and IMT/lumen ratio at age 17 and 21 years were assessed by ultrasound using a linear 12-MHz transducer (Vivid7, GE Medical, Chicago, Illinois) (18). Measurements were repeated for 3 different cardiac cycles and averaged. Interobserver variability for cIMT was assessed in a separate sample of 25 young adults (coefficient of variation: 4.4 ± 2.2%). Lumen diameter was calculated as external vessel diameter: (2 × cIMT). Mean circumferential wall stress (CWS; kPa) was calculated as: (mean arterial pressure × lumen diameter)/(2 × cIMT) (19).

#### Radial artery structure and stress using UHFUS

In a subsample of 172 participants (67% female) recruited at age 21 years, radial IMT and individual intimal and medial thicknesses were assessed by UHFUS using a linear MS700 30- to 70-MHz transducer (Vevo 2100, Fujifilm Visualsonics, Amsterdam, the Netherlands) with a scanning frequency of 50 MHz. Interobserver variability was assessed in a separate cohort of 10 young adults (coefficient of variation: 8.6 ± 3.7% and 8.5 ± 5.4% for radial IMT and medial thickness, respectively). Full details of the method and reproducibility were published elsewhere (12).

### Cardiovascular risk factors

#### Body size and composition

At all ages, participant height (meters) was measured using a stadiometer (SECA 213, Birmingham, United Kingdom) and body mass (kilogram) using electronic weighing scales (Marsden M-110, Rotherham, United Kingdom). Body mass index (BMI) was calculated as body mass/height^2^. Body composition at ages 9, 11, 13, 15, and 17 years was assessed using a Lunar Prodigy DEXA scanner (GE Medical Systems, Madison, Wisconsin), whereas body composition at age 21 years was assessed using cardiac magnetic resonance (1.5T Avanto, Siemens Medical Solutions, New York, New York); details of both procedures were published previously (20,21). Fat-free mass (FFM) was calculated as body mass – fat mass (FM).

#### Blood pressure

BP was measured on up to 6 occasions between the ages of 9 and 21 years (details in the Supplemental Appendix).

#### Metabolic risk factors

Blood samples (ALSPAC@17 fasting; ALSPAC@21 >4 to 6 h post-meal) were taken using standard procedures, immediately spun, separated, and frozen at –80 °C. Lipid profile (low-density lipoprotein cholesterol, high-density lipoprotein cholesterol, and triglycerides), glucose, insulin, and C-reactive protein were measured as described previously (22).

#### Lifestyle risk factors

Socioeconomic class, smoking status, physical activity, dietary intake, and exposure to passive smoke exposure were included in analyses as potential confounders (details in the Supplemental Appendix).

### Statistical analyses

Continuous data were summarized as mean ± SD or median (interquartile range) if skewed. In cross-sectional analyses at 17 and 21 years, multiple linear regression analyses were used to assess associations between a range of well-established CV risk factors and cIMT, and independent predictors were identified. All variables were converted to z-scores to allow comparisons between exposures. All multivariable models were inspected for collinearity and a variance inflation factor <5 was chosen as the threshold for acceptance. Multiple imputations (20 imputed datasets) were used to account for missing variables before analyses. Details of missing data at ages 17 and 21 years are shown in Supplemental Tables 2 and 3. To evaluate the effect of cumulative exposure to risk factors throughout adolescence and cIMT at age 17 years, we implemented latent class growth mixture modeling (LCGMM) analysis as previously described (23). By LCGMM, we characterized discrete patterns of FFM, FM, and systolic BP alterations from age 9 to 17 years. One to 5 classes were tested in LCGMM models, and selection was based on log-likelihood and Bayes information criterion indexes as well as posterior classification probabilities. Random intercept and slope(s) variance models were used in LCGMM analysis to flexibly model between-subjects heterogeneity in longitudinal changes in variables of interest. Linear, quadratic, and cubic specifications for the within-subject response of FFM, FM, and systolic BP as a function of age were tested (Supplemental Tables 4 to 6). z-Scores were used to enhance numeric stability and minimize differences between sexes. To account for interindividual growth differences, repeated measures were indexed to height squared. To test the effect of differing body compositions on arterial structural remodeling, multiple regression models were used to relate different ratios of FFM and FM to lumen diameter, cIMT, IMT/lumen ratio, and vessel CWS. Quartiles of FFM were assessed against each dependent variable while adjusting for FM and all other covariates, and then repeated using quartiles of FM. All analyses at age 21 years included additional adjustment for genetic recall groups due to the recall-by-genotype design of this sample, as described in detail elsewhere (17). Cross-sectional analyses were conducted using SPSS (version 22, IBM, Armonk, New York) or Stata (version 15, StataCorp, College Station, Texas). The “lcmm” package in RStudio (version 1.1.414, Boston, Massachusetts) was used in LCGMM analysis. All tests were 2-sided. A priori, we planned to draw conclusions based on effect estimates and their confidence intervals (CIs), rather than statistical tests using an arbitrary p value cutoff. For example, given 2 effects with the same point estimate—one with narrow CIs, the other with wider CIs that may even include the null—we described both as showing the same effect. However, one is more imprecisely estimated and should be treated with more caution until replicated in a larger sample. The use of “positive” and “inverse” throughout the text refer to directional association rather than clinical implication.

## Results

### Participant characteristics

Participants in ALSPAC@17 were mean age of 17.8 ± 0.4 years and were 56% female, whereas those in ALSPAC@21 were on average 20.9 ± 1.0 years of age and 66% female. Other characteristics are shown in Table 1.

**Table 1:** Participant Characteristics

	ALSPAC at 17 Years		ALSPAC at 21 Years	
	n	Mean ± SD / Median (IQR)	n	Mean ± SD / Median (IQR)
Age (yrs)	5,215	17.8 ± 0.4	436	20.9 ± 1.0
Male/female (%)	5,215	44/56	436	34/66
Height (m)	5,068	1.71 ± 0.09	436	1.71 ± 0.09
BMI (kg/m^2^)	5,062	22.0 (20.1–24.6)	436	23.4 (20.7–26.5)
Mass (kg)	5,067	64.8 (57.4–73.9)	436	68.6 (59.2–80.3)
FFM (kg)	4,849	43.0 (37.2–54.1)	407	51.8 (45.6–62.2)
FM (kg)	4,849	16.4 (10.7–23.4)	407	15.0 (10.7–21.1)
SBP (mm Hg)	4,658	116 ± 10	431	117 ± 10
PP (mm Hg)	4,658	52 ± 9	431	50 ± 9
DBP (mm Hg)	4,658	64 ± 6	431	67 ± 7
MAP (mm Hg)	4,658	81 ± 8	431	84 ± 7
Carotid lumen diameter (mm)	4,543	6.11 ± 0.40	435	5.31 ± 0.40
Carotid IMT (mm)	4,619	0.48 ± 0.04	435	0.46 ± 0.04
Radial lumen diameter (mm)	―	―	172	1.88 ± 0.37
Radial IMT (mm)	―	―	172	0.15 ± 0.03
MT	―	―	172	0.09 ± 0.03
IT	―	―	172	0.07 ± 0.01
LDL-C (mmol/l)	3,284	2.11 ± 0.61	384	1.85 ± 0.73
HDL-C (mmol/l)	3,284	1.27 ± 0.30	384	1.03 ± 0.35
Triglycerides (mmol/l)	3,284	0.75 (0.60–0.98)	384	0.67 (0.49–0.91)
Glucose (mmol/l)	3,284	5.00 (4.76–5.25)	385	3.80 (3.35–4.17)
Insulin (μU/ml)	3,230	6.77 (4.89–9.85)	385	7.27 (5.30–10.45)
CRP (mg/l)	3,284	0.55 (0.28–1.35)	378	0.77 (0.30–2.09)
Smoking status (% ever smoked)	4,191	48.8	423	18.2
Passive smoke exposure (% >1 h day)	3,423	44	―	―
Daily dietary intake (kcal)	4,561	2,238 ± 189	―	―
Physical activity				
Counts/min	1,943	479 ± 178	―	―
% <2× weeks	―	―	436	43.1
Highest household social class	4,336		392	
I	178	4.1	26	6.6
II	1,076	24.8	122	31.1
III (non-manual)	1,155	26.6	112	28.6
III (manual)	1,155	26.6	88	22.4
IV	607	14.0	36	9.2
V	1,60	3.7	6	1.5

ALSPAC = Avon Longitudinal Study of Parents and Children; BMI = body mass index; CRP = C-reactive protein; DBP = diastolic blood pressure; FFM = fat-free mass; FM = fat mass; HDL-C = high-density lipoprotein cholesterol; IMT = intima-media thickness; IT = intima thickness; LDL-C = low-density lipoprotein cholesterol; MAP = mean arterial pressure; MT = media thickness; SBP = systolic blood pressure.

### Cross-sectional associations between CV risk factors and cIMT

At age 17 years, FFM (mean difference in cIMT per 1-SD increase in exposure: 0.009 mm [95% CI: 0.008 to 0.010]; systolic BP difference: 0.007 mm [95% CI: 0.006 to 0.009]; physical activity level difference: 0.007 mm [95% CI: 0.005 to 0.008]; and dietary intake difference: 0.002 mm [95% CI: 0.004 to 0.006]) was found to be the modifiable risk factor positively correlated with cIMT, whereas FM (difference: −0.003 mm; 95% CI: −0.004 to −0.002) and C-reactive protein (difference: −0.002 mm; 95% CI: −0.004 to −0.001) were found to be negatively correlated (Table 2). Positive relationships were also seen for the nonmodifiable risk factors of male sex and height (Table 2). In a multivariable regression model that incorporated all significant univariate predictors, only male sex, FFM, and systolic BP remained positively associated with cIMT, whereas FM remained negatively associated (Table 2). Regression models conducted without multiple imputation were consistent with these findings (Supplemental Table 7). Results from the recall cohort at age 21 years (both in the cohort as a whole and in the subset with only UHFUS measures) showed similar effect sizes as those observed at age 17 years, albeit with wider 95% CIs due to the limited numbers of participants (Supplemental Table 8).

**Table 2:** Multivariable Linear Regression Analysis Identifying Independent Associations Between Cardiovascular Risk Factors and cIMT at Age 17 Years

	Mean Change in cIMT (mm) per 1-SD Increase in Exposure Variable (95% CI)	p Value
Univariate predictors		
Age	−0.001 (−0.02 to 0.001)	0.486
Height	0.008 (0.006 to 0.009)	<0.001
Male	0.007 (0.006 to 0.009)	<0.001
FFM	0.009 (0.008 to 0.010)	<0.001
FM	−0.003 (−0.004 to −0.002)	<0.001
SBP	0.007 (0.006 to 0.009)	<0.001
DBP	−0.001 (−0.002 to −0.000)	0.166
LDL-C	0.000 (−0.002 to 0.001)	0.629
HDL-C	−0.001 (−0.003 to 0.002)	0.575
Triglycerides	−0.001 (−0.003 to 0.001)	0.306
Glucose	0.001 (−0.001 to 0.003)	0.392
Insulin	0.000 (−0.002 to 0.002)	0.920
CRP	−0.002 (−0.004 to −0.001)	0.010
Smoking (ever)	0.000 (−0.003 to 0.003)	0.835
Physical activity	0.007 (0.005 to 0.008)	<0.001
Dietary intake	0.004 (0.002 to 0.006)	0.002
Multivariable model		
Male	0.004 (0.002 to 0.007)	0.002
Height	0.002 (0.000 to 0.004)	0.096
FFM	0.007 (0.004 to 0.010)	<0.001
FM	−0.003 (−0.004 to −0.001)	0.001
SBP	0.005 (0.004 to 0.008)	<0.001
CRP	−0.002 (−0.003 to 0.000)	0.114
Physical activity	0.000 (−0.002 to 0.002)	0.980
Dietary intake	0.002 (−0.001 to 0.004)	0.216

Multiple imputation was used to account for missing variables of interest.

CI = confidence interval; cIMT = carotid intima-media thickness; other abbreviations as in Table 1.

### Cumulative exposure to CV risk factors throughout adolescence and cIMT

Based on the strong positive relationships between FFM and systolic BP with cIMT, and the negative relationship between FM and cIMT, longitudinal analyses were used to test relationships between cumulative exposure to these risk factors throughout adolescence and cIMT at age 17 years. To assess the impact of increased lean mass per se versus that of altering adiposity levels per se, 3 distinct trajectories of FFM and FM were identified by LCGMM analysis (Supplemental Figures 1 and 2). For FFM, most participants were classified as low exposure (n = 2,134), whereas the remaining participants were middle (n = 514) and high exposure (n = 1,913), respectively. Similarly, for FM changes, participants were classified as low (n = 905), middle (n = 2,250), and high exposures (n = 1,406). In fully adjusted models, including the aforementioned, covariates plus socioeconomic status, physical activity, smoking, exposure to second-hand smoke, and dietary intake, high exposure to FFM throughout adolescence was associated with greater cIMT at 17 years (mean difference in cIMT between low- and high-exposure groups: 0.008 mm; 95% CI: 0.002 to 0.015; p = 0.010) (Table 3). No interaction between sex and FFM exposure on cIMT at 17 years was observed (p = 0.245). By levels of adiposity, participants with the highest FM exposure (i.e., fattest participants) were found to have lower cIMT at 17 years (difference: −0.005 mm; 95% CI: −0.010 to 0.000; p = 0.037). In addition to cumulative exposures to different body compositions, 3 groups of cumulative exposure to systolic BP were also identified as follows: low (n = 3,580), middle (n = 556), and high systolic BP (n = 421) (Supplemental Figure 3). Having high systolic BP across adolescence was associated with increased cIMT at 17 years (mean difference in cIMT in high-exposure group compared with low-exposure group: 0.007 mm; 95% CI: 0.001 to 0.012; p = 0.017). Findings from regression models without imputation for missing values were consistent with these results (Supplemental Table 9).

**Table 3:** Longitudinal Exposure to Differing Body Compositions and SBP Throughout Adolescence and cIMT at Age 17 Years

	Model 1		Model 2		Model 3	
	Effect Estimate (95% CI)	p Value	Effect Estimate (95% CI)	p Value	Effect Estimate (95% CI)	p Value
Absolute FFM						
Low	Reference	―	Reference	―	Reference	―
Middle	0.002 (−0.002 to 0.006)	0.351	0.002 (−0.002 to 0.006)	0.370	0.001 (−0.004 to 0.007)	0.629
High	0.014 (0.011 to 0.017)	< 0.001	0.008 (0.002 to 0.013)	0.006	0.008 (0.002 to 0.015)	0.010
Absolute FM						
Low	Reference	―	Reference	―	Reference	―
Middle	−0.001 (−0.004 to 0.002)	0.642	−0.004 (−0.001 to −0.007)	0.015	−0.003 (−0.007 to 0.000)	0.068
High	−0.010 (−0.014 to 0.006)	< 0.001	−0.005 (−0.010 to −0.001)	0.014	−0.005 (−0.010 to 0.000)	0.037
SBP						
Low	Reference	―	Reference	―	Reference	―
Middle	0.011 (0.007 to 0.015)	< 0.001	0.004 (0.001 to 0.008)	0.043	0.004 (−0.001 to 0.009)	0.100
High	0.008 (0.003 to 0.013)	0.001	0.007 (0.002 to 0.012)	0.003	0.007 (0.001 to 0.012)	0.017

FFM and FM were indexed to height squared to account for growth throughout adolescence. Model 1; Unadjusted. Model 2: Model 1 + adjustments for sex, height, SBP, LDL-C, and CRP. Model 3: Model 2 + adjustments for socioeconomic class, physical activity, smoking, exposure to passive smoking, and dietary intake. Absolute FFM model also adjusted for FM; absolute FM model for FFM; and SBP model for both FFM and FM. SBP trajectories were not adjusted for SBP at 17 years.

Abbreviations as in Tables 1 and 2.

### Cross-sectional associations between body composition and carotid artery structure and stress

**Figure 1:**
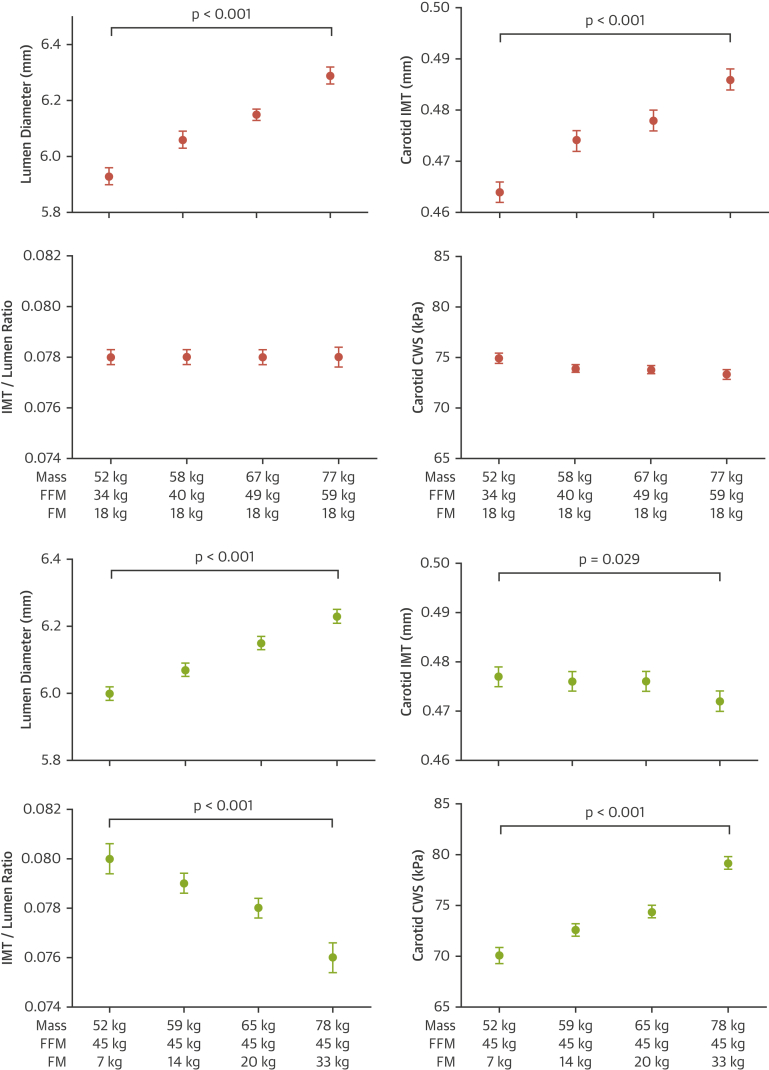
Relationship Between Changes in Body Mass Due to Increasing FFM or FM and Carotid Structure and Wall Stress at Age 17 Years Relationship between changes in body mass due to increasing fat-free mass (FFM) **(pink)** or fat mass (FM) **(green)** and carotid structure and wall stress at age 17 years. All models adjusted for age, sex, and height. The p value is for linear trend. **Error bars** denote 95% confidence intervals (CIs). CWS = circumferential wall stress; IMT = intima-media thickness.

Due to the opposite associations observed for FFM (positive) and FM (negative) with cIMT, we assessed differences in carotid artery remodeling with increasing FFM (i.e., growth), or with increased FM (i.e., adiposity). Growth was associated with equivalent increases in both cIMT and lumen diameter, which resulted in an unchanged IMT/lumen ratio and a preserved vessel CWS in the face of increased mean arterial pressure. In contrast, increasing adiposity was associated with an increased carotid lumen diameter, but a reduced cIMT, which resulted in a reduced IMT/lumen ratio and progressively increased CWS as levels of FM and mean arterial pressure increased (Figure 1). Similar results were observed at age 21 years, with elevated levels of FFM associated with similar increases in lumen diameter and cIMT, and therefore, preserved CWS, whereas increased FM showed a trend for increased lumen diameter, decreased IMT, and a progressive increase in CWS (Supplemental Figure 4).

### Associations between CV risk factors and individual intimal and medial layers within the radial artery

**Figure 2:**
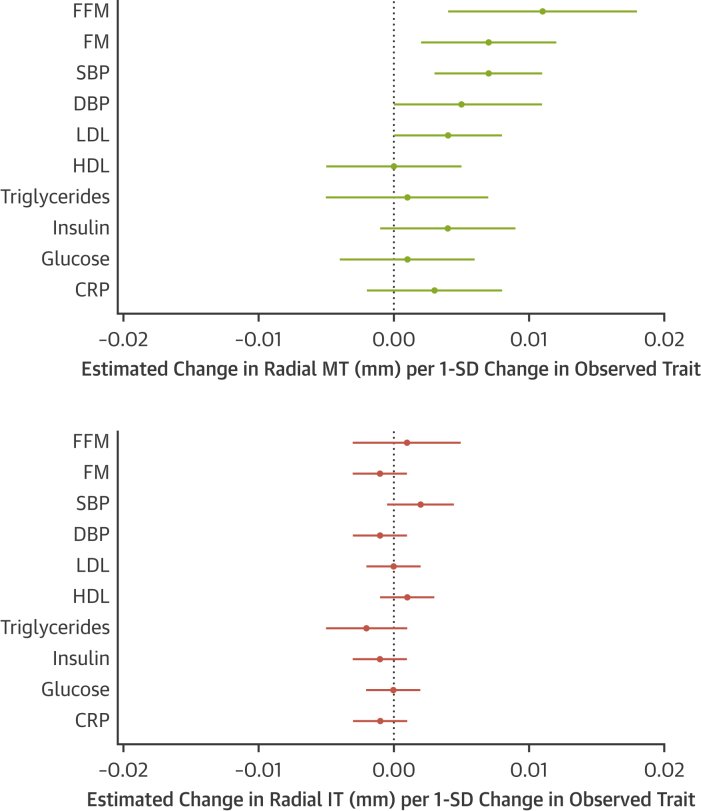
Relationship Between Cardiovascular Risk Factors and Radial Media and Intima Thicknesses Relationship between cardiovascular risk factors and individual radial media and intima thicknesses at age 21 years. All models adjusted for age, sex, and height. **Error bars** denote 95% CI. CRP = C-reactive protein; DBP = diastolic blood pressure; HDL = high-density lipoprotein cholesterol; LDL = low-density lipoprotein cholesterol; MT = media thickness; SBP = systolic blood pressure; other abbreviations as in Figure 1.

Radial IMT at age 21 years showed similar relationships to FFM and FM as those observed for cIMT at both 17 and 21 years (Supplemental Figure 5). When assessing the impact of growth and adiposity on intimal and medial layers separately, increases in radial IMT were attributable to an increase in media thickness, with intimal thickness unchanged in relation to growth (Supplemental Figure 6). Similar to cIMT at this age, we found little evidence of a relationship between increased adiposity and thickening in either the medial or intimal layer (Supplemental Figure 6). In models adjusted for age, sex, and height, media thickness was found to be associated with changes in body composition (FFM and FM) and BP (systolic BP and diastolic BP) (Figure 2). In contrast to findings in the medial layer, we did not observe any evidence of a relationship between any measure of body composition (FFM or FM), BP, lipids (low-density lipoprotein cholesterol, high-density lipoprotein cholesterol, triglycerides), glucose homeostasis (glucose, insulin), or inflammatory (C-reactive protein) risk factors with intima thickness (Figure 2).

## Discussion

Our study suggested that subtle differences in cIMT commonly observed in adolescents and young adults are likely to represent arterial remodeling in response to increased levels of FFM and BP, rather than being an early marker of intimal disease. A number of findings supported this hypothesis. First, we showed that increases in FFM were independently associated with increases in both carotid cIMT and lumen diameter, thereby maintaining the arterial IMT/lumen ratio and CWS despite increases in BP and vessel diameter. Second, using longitudinal measures of body composition collected between the ages of 10 and 17 years, we found that those with the highest cumulative exposure to FFM and systolic BP throughout adolescence had the greatest cIMT at age 17 years, whereas those with the highest relative adiposity (i.e., highest absolute FM) displayed the opposite. Finally, using novel UHFUS technology, we demonstrated that FFM-related changes in cIMT were mirrored in the radial artery. These changes occurred in the medial layer alone and were associated with body composition and BP. We also found little evidence of an association between intimal thickness and any measured CV risk factor.

cIMT is a well-established risk marker for atherosclerosis and although its use is not recommended in clinical guidelines for risk stratification―has been shown to predict future cardiovascular events independently of traditional risk factors in adults (2,23,24). Due to a lack of clinical events in the young, studies in children and adolescents, such as a recent study in metabolically healthy and unhealthy individuals ages 6 to 17 years of age (25), commonly use cIMT as a surrogate endpoint linking early CVD risk factor exposure to subclinical atherosclerotic disease (3, 4, 5, 6). However, whether cIMT truly reflects the earliest indication of intimal disease or is largely a reflection of other growth-related changes in arterial morphology, has never been rigorously investigated. Using a large and extensively phenotyped longitudinal cohort, we showed in both cross-sectional and prospective analyses that higher FFM was the predominant factor that explained the subtle increases in cIMT in young adulthood. These findings supported recently published work that related body composition to numerous subclinical markers of disease in the Southampton Women’s Study, in which the lean mass index, but not the FM index, was found to predict cIMT in healthy 8- to 9-year-old children (10), as well as in a number of smaller adult studies, where FFM was found to explain much of the obesity-related variance in cIMT (8,9). Increases in FFM in the present study were found to be positively associated with both carotid lumen diameter and cIMT, which resulted in the maintenance of the IMT/lumen ratio and a preservation of vessel CWS when increases in body mass consisted of lean tissues. Use of UHFUS in the radial artery identified these changes as being confined to the medial layer of the vessel wall alone. This suggested that subtle changes in cIMT often attributed to excess adiposity in the young were likely to represent an anatomical remodeling response to an underlying accumulation of FFM and an increase in SBP, rather than the development of intimal disease in relation to excess FM and its associated cardiometabolic risk factors.

The relationship between FFM and cIMT at this age is likely partially mediated by increases in systolic BP that accompany accumulation of FFM. It is well-established that the high metabolic demands of lean tissue accumulation necessitate a chronic increase in blood flow. This requirement is met through stroke volume−driven increases in cardiac output (17), which, in turn elevates BP. Previous research in adults (26,27) showed systolic BP to be the component of BP most strongly associated with cIMT. We replicated this observation in young people and expanded on it by identifying the medial layer as the site of thickening when FFM increases at this age. In contrast to FFM, neither FM nor its related CV risk factors (diastolic BP, lipids, glucose) were associated with vessel wall thickening once FFM was accounted for, with increasing FM levels instead associated with decreased cIMT at age 17 years. Consequently, participants with greater levels of adiposity had progressive increases in vessel CWS as both diameter and MAP were increased and wall thickness was decreased. Although not tested in this study, this cumulative exposure to increased wall stress likely increased fractional collagen (rather than elastin) engagement within the vessel wall, leading to progressive stiffening of the major elastic arteries over time. These changes, combined with high levels of circulating metabolic risk factors, might confer long-term damage to the vasculature. Overall, our data suggested that the subtle increases in cIMT observed at this age in association with FFM may potentially represent a protective remodeling response within the vessel media, rather than the initial signs of atherosclerosis.

It is important to note that our findings were derived from a young predominantly healthy population cohort and did not imply that cIMT is an unsuitable surrogate marker for subclinical atherosclerotic disease in other clinical populations, either young or old. Although we propose that subtle changes in cIMT at this age were likely to reflect FFM-related arterial remodeling, several studies suggested that more pronounced arterial wall thickening in older or unhealthy populations with more complex risk factor profiles were likely to be pathological. First, although cIMT was found to be predominantly related to body size (BMI and/or waist/hip ratio) and BP (SBP and pulse pressure) in adolescence (11 to 17 years) in a study of 635 healthy adolescents and young adults taking part in the Muscatine Offspring Study, additional independent associations for numerous other established CVD risk factors (total cholesterol, low-density lipoprotein cholesterol, triglycerides, and glycosylated hemoglobin) began to emerge when the same analyses were carried out in an older age group (18 to 34 years) with more pronounced changes in cIMT (28). Second, both genetic and observational studies from the slightly older Cardiovascular Risk in Young Finns cohort reported positive relationships between longitudinal measures of BMI and changes in cIMT at age 24 to 39 years (29). This suggested that extended cumulative exposure to CV risk factors (e.g., elevated lipids, inflammation, and so on) might be critical in the development of arterial damage in later adulthood. Finally, using UHFUS in children with chronic kidney disease, our group previously demonstrated differential remodeling of the radial artery wall compared with healthy individuals. This pathological response was characterized by pronounced medial thickening in tandem with elevated serum phosphate levels that resolved following renal transplantation, which suggested an adverse influence of the uremic milieu on vessel structure (14). Future studies using UFHUS in other high-risk adolescent groups (e.g., familial hypercholesterolemia or diabetes) might help to highlight the underlying wall layers driving IMT in different pathological states.

### Study Strengths and limitations

The observational nature of our findings prevented us from inferring causal relationships between exposures and outcomes. However, our findings built upon a recent Mendelian randomization study of this cohort that did not observe a causal relationship between BMI and cIMT (17). Access to one of the world’s largest and most extensively phenotyped longitudinal birth cohorts (ALSPAC) allowed us to conduct both cross-sectional and prospective analyses while adjusting for a wide range of sociodemographic, health-related, and lifestyle factors, thereby minimizing the potential for undetected confounding. Although a higher response rate from females in ALSPAC@21 might have affected results, we attempted to account for this in statistical models, and our findings in this group were consistent with those at age 17 years. The use of a novel UHFUS technique in a subset of these participants allowed us to explore the anatomical changes underlying increases in IMT that could not be detected using traditional ultrasound techniques. A potential limitation of UHFUS was the limited penetration depth of the ultrasound signal (approximately 1 cm), which required us to use a peripheral radial artery rather than the gold standard carotid artery for these tests. Research using UHFUS in older participants showed radial artery measures offered the same predictive risk for coronary heart disease compared with carotid measures (13). Although the possibility exists that different vessels might not respond to risk factors in the same way in the young, the virtually identical effect sizes between FFM and both cIMT (at age 17 and 21 years) and radial IMT (at age 21 years) suggested similar remodeling responses (Supplemental Figure 5, Supplemental Table 8). Although the preservation of CWS suggested that this remodeling was predominantly adaptive in nature, we could not rule out the presence of an early pathological response to increased BP because of its known impact on future disease risk. Finally, although systematic differences across the follow-up period of the study were taken into consideration by age and/or study year−specific values in LCGMM analysis, the potential for residual bias in BP analyses due to the different methodologies and/or equipment used at each clinic visit could not be excluded. In addition, despite efficient latent class separation and satisfactory fitting to observed data for measures of FFM, FM, and systolic BP, residual within-group variability that was not accounted for might still exist.

## Conclusions

These findings suggest that subtle changes in cIMT in young healthy populations may predominantly represent physiological adaptation to differing levels of FFM as opposed to evidence of intimal disease and signs of early subclinical atherosclerosis (Central Illustration). As a result, increases in cIMT should be interpreted with caution before adulthood. Other vascular measures may potentially be more appropriate for the identification of early arterial disease at this age.

Perspectives **COMPETENCY IN MEDICAL KNOWLEDGE:** Subtle changes in cIMT in young healthy populations appear to be confined to the medial layer and may predominantly represent physiological adaptation to differing levels of FFM mass rather than evidence of intimal disease and signs of early subclinical atherosclerosis.

**TRANSLATIONAL OUTLOOK:** These findings are derived from a young and healthy population cohort free from established clinical disease. Additional studies are needed to establish whether the evolution of cIMT differs in individuals with differing risk factor profiles (e.g., young people with diabetes, familial hypercholesterolemia, and so on). Until such time, increases in cIMT should be interpreted with caution before adulthood.

**Central Illustration:**
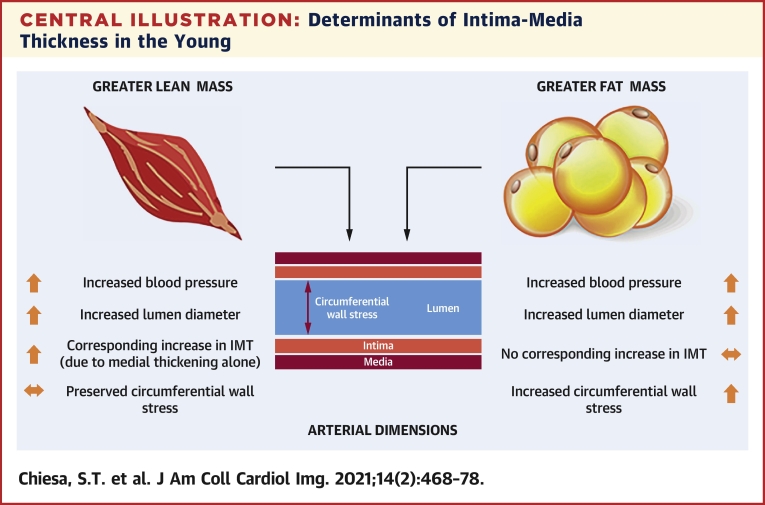
Determinants of Intima-Media Thickness in the Young Subtle increases in intima-media thickness in a young healthy population appear predominantly medial-driven, are associated with increased fat-free mass as opposed to fat mass, and act to maintain vessel circumferential wall stress in the face of increasing blood pressure.

## Author Disclosures

This research was funded through grants from the British Heart Foundation (RG/10/004/28240, PG/06/145, and CS/15/6/31468), University of Bristol, and UK Medical Research Council and Wellcome Trust (102215/2/13/2, MC_UU_12013/1–9, 096989/Z11/Z, and 086676/7/08/Z). Dr. Deanfield was supported by the British Heart Fund. Dr. Timpson was supported by the University of Bristol NIHR Biomedical Research Centre (BRC-1215-20011) and the MRC Integrative Epidemiology Unit (MC_UU_12013/3). Drs. Davey Smith and Lawlor work in the Medical Research Council Integrative Epidemiology Unit at the University of Bristol, which is supported by the Medical Research Council (MC_UU_00011/1 and MC_UU_00011/6). Dr. Lawlor has received support from Medtronic and Roche Diagnostics. All other authors have reported that they have no relationships relevant to the contents of this paper to disclose.

## References

[1] JBS3 Board (2014). Joint British Societies’ consensus recommendations for the prevention of cardiovascular disease (JBS3). Heart.

[2] Polak J.F., Pencina M.J., Pencina K.M., O’Donnell C.J., Wolf P.A., D’Agostino R.B. (2011). Carotid-wall intima–media thickness and cardiovascular events. N Engl J Med.

[3] Meyer A.A., Kundt G., Steiner M., Schuff-Werner P., Kienast W. (2006). Impaired flow-mediated vasodilation, carotid artery intima-media thickening, and elevated endothelial plasma markers in obese children: the impact of cardiovascular risk factors. Pediatrics.

[4] Karpoff L., Vinet A., Schuster I. (2009). Abnormal vascular reactivity at rest and exercise in obese boys. Eur J Clin Invest.

[5] Yilmazer M.M., Tavli V., Carti Ö.U. (2010). Cardiovascular risk factors and noninvasive assessment of arterial structure and function in obese Turkish children. Eur J Pediatr.

[6] Peña A.S., Wiltshire E., MacKenzie K. (2006). Vascular endothelial and smooth muscle function relates to body mass index and glucose in obese and nonobese children. J Clin Endocrinol Metab.

[7] Ayer J., Charakida M., Deanfield J.E., Celermajer D.S. (2015). Lifetime risk: childhood obesity and cardiovascular risk. Eur Heart J.

[8] Moreno M., Puig J., Moreno-Navarrete J.M. (2015). Lean mass, and not fat mass, is an independent determinant of carotid intima media thickness in obese subjects. Atherosclerosis.

[9] Kozakova M., Palombo C., Paterni M. (2008). Body composition and common carotid artery remodeling in a healthy population. J Clin Endocrinol Metab.

[10] Sletner L., Mahon P., Crozier S.R. (2018). Childhood fat and lean mass. Arterioscler Thromb Vasc Biol.

[11] Osika W., Dangardt F., Grönros J. (2007). Increasing peripheral artery intima thickness from childhood to seniority. Arterioscler Thromb Vasc Biol.

[12] Eklund C., Friberg P., Gan L.-M. (2012). High-resolution radial artery intima-media thickness and cardiovascular risk factors in patients with suspected coronary artery disease – comparison with common carotid artery intima-media thickness. Atherosclerosis.

[13] Eklund C., Omerovic E., Haraldsson I., Friberg P., Gan L.-M. (2014). Radial artery intima-media thickness predicts major cardiovascular events in patients with suspected coronary artery disease. Eur Hear J Cardiovasc Imaging.

[14] Dangardt F., Charakida M., Chiesa S. (2018). Intimal and medial arterial changes defined by ultra-high-frequency ultrasound: response to changing risk factors in children with chronic kidney disease. PLoS One.

[15] Fraser A., Macdonald-Wallis C., Tilling K. (2013). Cohort profile: the Avon Longitudinal Study of Parents and Children: ALSPAC mothers cohort. Int J Epidemiol.

[16] Boyd A., Golding J., Macleod J. (2013). Cohort profile: the ’Children of the 90s’--the index offspring of the Avon Longitudinal Study of Parents and Children. Int J Epidemiol.

[17] Wade K.H., Chiesa S.T., Hughes A.D. (2018). Assessing the causal role of body mass index on cardiovascular health in young adults: Mendelian randomization and recall-by-genotype analyses. Circulation.

[18] Frysz M., Deere K., Lawlor D.A., Benfield L., Tobias J.H., Gregson C.L. (2016). Bone mineral density is positively related to carotid intima-media thickness: findings from a population-based study in adolescents and premenopausal women. J Bone Miner Res.

[19] Bussy C., Boutouyrie P., Lacolley P., Challande P., Laurent S. (2000). Intrinsic stiffness of the carotid arterial wall material in essential hypertensives. Hypertension.

[20] Yu H., McKenzie C.A., Shimakawa A. (2007). Multiecho reconstruction for simultaneous water-fat decomposition and T2* estimation. J Magn Reson Imaging.

[21] Riddoch C.J., Leary S.D., Ness A.R. (2009). Prospective associations between objective measures of physical activity and fat mass in 12-14 year old children: the Avon Longitudinal Study of Parents and Children (ALSPAC). BMJ.

[22] Falaschetti E., Hingorani A.D., Jones A. (2010). Adiposity and cardiovascular risk factors in a large contemporary population of pre-pubertal children. Eur Heart J.

[23] O’Leary D.H., Polak J.F., Kronmal R.A., Manolio T.A., Burke G.L., Wolfson S.K. (1999). Carotid-artery intima and media thickness as a risk factor for myocardial infarction and stroke in older adults. N Engl J Med.

[24] Bots M.L., Hoes A.W., Koudstaal P.J., Hofman A., Grobbee D.E. (1997). Common carotid intima-media thickness and risk of stroke and myocardial infarction: the Rotterdam Study. Circulation.

[25] Zhao M., López-Bermejo A., Caserta C.A. (2019). Metabolically healthy obesity and high carotid intima-media thickness in children and adolescents: International Childhood Vascular Structure Evaluation Consortium. Diabetes Care.

[26] Zanchetti A., Crepaldi G., Bond M.G. (2001). Systolic and pulse blood pressures (but not diastolic blood pressure and serum cholesterol) are associated with alterations in carotid intima-media thickness in the moderately hypercholesterolaemic hypertensive patients of the Plaque Hypertension Lipid Lowering Italian Study. PHYLLIS study group. J Hypertens.

[27] Ferreira J.P., Girerd N., Bozec E. (2016). Intima-media thickness is linearly and continuously associated with systolic blood pressure in a population-based cohort (STANISLAS Cohort Study). J Am Heart Assoc.

[28] Dawson J.D., Sonka M., Blecha M.B., Lin W., Davis P.H. (2009). Risk factors associated with aortic and carotid intima-media thickness in adolescents and young adults. J Am Coll Cardiol.

[29] Kivimaki M., Smith G.D., Timpson N.J. (2008). Lifetime body mass index and later atherosclerosis risk in young adults: examining causal links using Mendelian randomization in the Cardiovascular Risk in Young Finns study. Eur Heart J.

