# Supplementary material for: Determinants of Intima-Media Thickness in the Young: The ALSPAC Study
Source: JACC Cardiovasc Imaging. 2021 Feb;14(2):468–78. doi: 10.1016/j.jcmg.2019.08.026 (PMC7851110; doi:10.1016/j.jcmg.2019.08.026)
Supplement: Supplemental Data [file mmc1.docx]

**Supplemental Methods**

***ALSPAC cohort***

Full details of the ALSPAC cohort and study design are available at the ALSPAC website (<http://www.alspac.bris.ac.uk>). This website also contains details of all data that is available through a fully searchable data dictionary and online variables search tool (http://www.bris.ac.uk/alspac/researchers/data-access/data-dictionary). Briefly, 14,541 pregnant women resident in a defined area of the South West of England, with an expected delivery date of 1st of April 1991 - 31st of December 1992 were enrolled to the cohort. Of these, 13,988 live-born children who were still alive 1 year later have been followed-up to date with regular questionnaires and clinical measures, providing behavioural, lifestyle and biological data.

***Blood Pressure Measurements***

At all of the clinics, systolic (SBP) and diastolic (DBP) blood pressure were measured twice with the participants at rest using the appropriate cuff size for the upper arm circumference, and the mean of each was recorded. BP was measured in the seated position using a Dinamap 9301 Vital Signs Monitor at the 9, 11, and 15-year clinics; a Dinamap 8100 Vital Signs Monitor at the 13-year clinic; an Omron 705-IT at the 17 year clinic; and in the supine position using an Omron M6 at the 21 year clinic. Pulse pressure (PP) was calculated as SBP – DBP, and mean arterial pressure (MAP) was calculated as DBP + (PP/3).

***Lifestyle Risk Factors***

Highest household occupation was used to assign participants a household social class, using the 1991 British Office of Population Census Statistics classification(1). Passive smoke exposure during childhood (< 1hr day / > 1hr day) and participant smoking status at both 17 and 21 (never / ever) were ascertained by questionnaires. Predicted calorie intake was calculated as previously described(2). At age 17, the most recent record of physical activity was defined as the average counts per minute (CPM) recorded over 7 days using an MTI Actigraph AM7164 2.2 accelerometer in a subsample of participants at the ALSPAC 15-year clinic. At age 21, physical activity was obtained via questionnaire and weekly exercise was categorised as either “never/rarely/<2 times a week” or “≥2 times a week”.

**Supplemental References**

1. Redfern P. Office of Population Censuses and Surveys. Encyclopedia of Statistical Sciences. John Wiley & Sons, Inc.; 2013. p. 1–5.

2. Anderson EL., Tilling K., Fraser A., et al. Estimating Trajectories of Energy Intake Through Childhood and Adolescence Using Linear-Spline Multilevel Models. Epidemiology 2013;24(4):507–15.

**Supplemental Table 1: Comparison of ALSPAC@17 characteristics in participants who were recalled for follow-up analysis at 21 years vs. those who were not**

|  | **Not Recalled @ 21** | **Recalled @ 21** |  |
| --- | --- | --- | --- |
| **Variable** | **Mean ± SD / Median (IQR)** | **Mean ± SD / Median (IQR)** | **p-value for difference** |
| Age (years) | 17.8 ± 0.4 | 17.8.9 ± 0.5 | 0.135 |
| Sex (% male/female) | 44/56 | 34/66 | < 0.001 |
| Height (m) | 1.71 ± 0.09 | 1.69 ± 0.09 | 0.001 |
| BMI (kg/m^2^) | 21.9 (20.1, 24.6) | 22.2 (20.2, 25.1) | 0.545 |
| Mass (kg) | 64.8 (57.5, 73.9) | 64.6 (55.8, 73.3) | 0.194 |
| FFM (kg) | 43.3 (37.2, 54.2) | 40.4 (36.8, 51.1) | < 0.001 |
| FM (kg) | 16.3 (10.7, 23.3) | 17.8 (12., 24.4) | 0.098 |
|  |  |  |  |
| SBP (mmHg) | 116 ± 10 | 116 ± 10 | 0.140 |
| PP (mmHg) | 52 ± 9 | 51 ± 9 | 0.012 |
| DBP (mmHg) | 64 ± 6 | 65 ± 6 | 0.001 |
| MAP (mmHg) | 85 ± 8 | 87 ± 8 | 0.005 |
| Carotid Lumen Diameter (mm) | 6.11 ± 0.40 | 6.08 ± 0.38 | 0.126 |
| Carotid IMT (mm) | 0.48 ± 0.05 | 0.47 ± 0.05 | 0.022 |
|  |  |  |  |
| LDL-c (mmol/l) | 2.10 ± 0.61 | 2.15 ± 0.60 | 0.263 |
| HDL-c (mmol/l) | 1.27 ± 0.30 | 1.25 ± 0.30 | 0.279 |
| Triglycerides (mmol/l) | 0.75 (0.60, 0.98) | 0.78 (0.60, 0.99) | 0.525 |
| Glucose (mmol/l) | 5.00 (4.77, 5.26) | 5.00 (4.74, 5.22) | 0.566 |
| Insulin (µU/ml) | 6.69 (4.87, 9.39) | 7.54 (5.71, 10.06) | 0.002 |
| CRP (mg/l) | 0.55 (0.28, 1.33) | 0.58 (0.28, 1.51) | 0.473 |
|  |  |  |  |
| Smoking Status (% ever smoked) | 48.8 | 50.0 | 0.254 |
| Physical Activity   - counts per minute | 481 ± 182 | 451 ± 137 | 0.035 |

**Supplemental Table 2: Data missingness for exposure variables in ALSPAC@17**

| **ALSPAC @ 17** | |
| --- | --- |
| **Exposure Variable** | **Missing observations (%)** |
| Age | 0 (0%) |
| Height | 101 (2.2%) |
| Sex | 0 (0%) |
| FFM | 185 (4.0%) |
| FM | 185 (4.0%) |
| SBP | 41 (0.9%) |
| DBP | 41 (0.9%) |
| LDL | 1579 (34.2%) |
| HDL | 1579 (34.2%) |
| Triglycerides | 1579 (34.2%) |
| Glucose | 1579 (34.2%) |
| Insulin | 1630 (35.3%) |
| CRP | 1579 (34.2%) |
| Physical Activity | 2816 (61.0%) |
| Dietary Intake | 58 (1.2%) |
| Smoking | 727 (15.7%) |
| Second-Hand Smoke Exposure | 1196 (28.7%) |
| Socioeconomic Status | 752 (16.3%) |

**Supplemental Table 3: Data missingness for exposure variables in ALSPAC@21**

| **ALSPAC @ 21** | |
| --- | --- |
| **Exposure Variable** | **Missing observations (%)** |
| Age | 0 (0%) |
| Height | 0 (0%) |
| Sex | 0 (0%) |
| FFM | 29 (6.7%) |
| FM | 29 (6.7%) |
| SBP | 5 (1.1%) |
| DBP | 5 (1.1%) |
| LDL | 52 (12.0%) |
| HDL | 52 (12.0%) |
| Triglycerides | 52 (12.0%) |
| Glucose | 51 (11.7%) |
| Insulin | 51 (11.7%) |
| CRP | 58 (13.3%) |

**Supplemental Table 4: Latent Class Growth Mixture Modelling selection of optimal number of classes for trajectories of FFM changes across the period of 9 to 17 years old**

| **Model** | **Nb. Latent classes** | **Polynomial degree** | **Log-Likelihood** | **BIC** | **% Participants per class** | **Mean posterior probabilities** | **Posterior probabilities > 0.7 (%)** |
| --- | --- | --- | --- | --- | --- | --- | --- |
| 1 | 1 | Linear | -18300.66 | 36651.9 | -100 | (n.a) | (n.a) |
| 2 |  | Quadratic | -18190.47 | 36465.25 | -100 | (n.a) | (n.a) |
| 3 |  | Cubic | -72420.83 | 144968.11 | -100 | (n.a) | (n.a) |
| 4 | 2 | Linear | -18049.87 | 36184.05 | (35.3/64.7) | (0.8896/0.9339) | (85.91/92.07) |
| 5 |  | Quadratic | -17826.43 | 35779.31 | (39.69/60.31) | (0.9254/0.9437) | (90.87/93.6) |
| 6 |  | Cubic | -17070.4 | 34317.83 | (100/0) | (0/NaN) | (0/NaN) |
| 7 | 3 | Linear | -17952.16 | 36022.35 | (6.9/37.84/55.27) | (0.7456/0.889/0.8542) | (57.59/85.41/84.01) |
| 8 |  | Quadratic | -17674.59 | 35517.79 | (46.85/11.24/41.92) | (0.8378/0.7718/0.9206) | (81.42/64.08/90.58) |
| 9 |  | Cubic | -17070.71 | 34369.04 | (100/0/0) | (0/NaN/NaN) | (0/NaN/NaN) |
| 10 | 4 | Linear | -17974.37 | 36075.19 | (0/55.4/6.11/38.49) | (NaN/0.4615/0.6363/0.8361) | (NaN/0/40.36/76.59) |
| 11 |  | Quadratic | -17629.1 | 35443.66 | (20.53/58.54/3.36/17.56) | (0.7508/0.8923/0.7771/0.7853) | (60.79/87.03/62.99/66.34) |
| 12 |  | Cubic | -47294.16 | 94841.22 | (100/0/0/0) | (0/NaN/NaN/NaN) | (0/NaN/NaN/NaN) |
| 13 | 5 | Linear | -17921.62 | 35994.99 | (36.72/51.87/0/4.6/6.81) | (0.7876/0.552/NaN/0.6599/0.7668) | (72.43/0/NaN/44.55/61.54) |
| 14 |  | Quadratic | -17574.73 | 35368.63 | (20.71/3.38/58.43/0.07/17.41) | (0.7516/0.7759/0.894/1/0.785) | (60.91/63.87/87.45/100/66.42) |
| 15 |  | Cubic | -54437.69 | 109170.43 | (100/0/0/0/0) | (0/NaN/NaN/NaN/NaN) | (0/NaN/NaN/NaN/NaN) |

Polynomial specification describes the within-subject functional association of longitudinal FFM changes with increasing age and includes linear, quadratic and cubic form to allow for curvilinear patterns. BIC, Bayes information criterion; n.a, non applicable, NaN, not calculated due to non-convergence of underlying models or suboptimal class separation (empty or 100% containing latent classes).

**Supplemental Table 5: Latent Class Growth Mixture Modelling selection of optimal number of classes for trajectories of FM changes across the period of 9 to 17 years old**

| **Model** | **Nb. Latent classes** | **Polynomial degree** | **Log-Likelihood** | **BIC** | **% Participants per class** | **Mean posterior probabilities** | **Posterior probabilities > 0.7 (%)** |
| --- | --- | --- | --- | --- | --- | --- | --- |
| 1 | 1 | Linear | -11952.05 | 23952.91 | -100 | (n.a) | (n.a) |
| 2 |  | Quadratic | -11660.37 | 23402.11 | -100 | (n.a) | (n.a) |
| 3 |  | Cubic | -10539.72 | 21201.49 | -100 | (n.a) | (n.a) |
| 4 | 2 | Linear | -11742.25 | 23565.86 | (23.32/76.68) | (0.8219/0.8346) | (71.89/86.07) |
| 5 |  | Quadratic | -11305.6 | 22733.24 | (70.24/29.76) | (0.8463/0.8552) | (86.58/77.58) |
| 6 |  | Cubic | -10055.69 | 20282.25 | (100/0) | (0/NaN) | (0/NaN) |
| 7 | 3 | Linear | -11696.75 | 23507.41 | (55.31/11.18/33.51) | (0.8482/0.8331/0.6879) | (80.85/73.3/48.3) |
| 8 |  | Quadratic | -11169.37 | 22501.48 | (38.43/45.19/16.39) | (0.8167/0.7552/0.8468) | (78.22/65.87/78.04) |
| 9 |  | Cubic | -10071.44 | 20362.56 | (100/0/0) | (0/NaN/NaN) | (0/NaN/NaN) |
| 10 | 4 | Linear | -11771.46 | 23664.98 | (0/0/86.04/13.96) | (NaN/NaN/0.3441/0.6938) | (NaN/NaN/0/51.78) |
| 11 |  | Quadratic | -11450.55 | 23080.1 | (100/0/0/0) | (0/NaN/NaN/NaN) | (0/NaN/NaN/NaN) |
| 12 |  | Cubic | -10254.19 | 20752.47 | (100/0/0/0) | (0/NaN/NaN/NaN) | (0/NaN/NaN/NaN) |
| 13 | 5 | Linear | -11771.46 | 23689.39 | (0/0/0/85.13/14.87) | (NaN/NaN/NaN/0.2964/0.6668) | (NaN/NaN/NaN/0/48.82) |
| 14 |  | Quadratic | -11450.55 | 23112.65 | (100/0/0/0/0) | (0/NaN/NaN/NaN/NaN) | (0/NaN/NaN/NaN/NaN) |
| 15 |  | Cubic | -11141.19 | 22567.15 | (100/0/0/0/0) | (0/NaN/NaN/NaN/NaN) | (0/NaN/NaN/NaN/NaN) |

Abbreviations as in Supplemental Table 4.

**Supplemental Table 6: Latent Class Growth Mixture Modelling selection of optimal number of classes for trajectories of SBP changes across the period of 9 to 17 years old**

| **Model** | **Nb. Latent classes** | **Polynomial degree** | **Log-Likelihood** | **BIC** | **% Participants per class** | **Mean posterior probabilities** | **Posterior probabilities > 0.7 (%)** |
| --- | --- | --- | --- | --- | --- | --- | --- |
| 1 | 1 | Linear | -23488.63 | 47027.87 | -100 | (n.a) | (n.a) |
| 2 |  | Quadratic | -23446.84 | 46952.72 | -100 | (n.a) | (n.a) |
| 3 |  | Cubic | -22340.16 | 44747.8 | -100 | (n.a) | (n.a) |
| 4 | 2 | Linear | -23447.23 | 46978.8 | (97.11/2.89) | (0.9604/0.7357) | (97.43/55.64) |
| 5 |  | Quadratic | -23410.61 | 46914 | (71.21/28.79) | (0.6859/0.7103) | (52.75/47.17) |
| 6 |  | Cubic | -22340.72 | 44782.65 | (0/100) | (NaN/1) | (NaN/100) |
| 7 | 3 | Linear | -23440.52 | 46999.13 | (8.84/84.4/6.76) | (0.4971/0.8283/0.6548) | (2.46/79.59/38.91) |
| 8 |  | Quadratic | -23399.42 | 46925.36 | (12.23/78.6/9.17) | (0.4922/0.7513/0.6326) | (1.95/70.43/35.31) |
| 9 |  | Cubic | -22340.41 | 44815.78 | (0/100/0) | (NaN/1/NaN) | (NaN/100/NaN) |
| 10 | 4 | Linear | -23431.68 | 47015.18 | (26.07/60.48/8.47/4.98) | (0.6465/0.7356/0.7198/0.7616) | (37.17/64.01/50.51/57.64) |
| 11 |  | Quadratic | -23422.3 | 47004.85 | (23.72/37.98/31.96/6.34) | (0.5216/0.4627/0.5781/0.6619) | (2.11/0/13.8/39.38) |
| 12 |  | Cubic | -1.00E+09 | 2000000169 | (100/0/0/0) | (0/NaN/NaN/NaN) | (0/NaN/NaN/NaN) |
| 13 | 5 | Linear | -23436.38 | 47058.31 | (3.37/29.65/52.73/7.89/6.37) | (0.3322/0.5096/0.5305/0.6611/0.7189) | (0/2.12/0/38.84/50.17) |
| 14 |  | Quadratic | -23433.25 | 47060.48 | (24.88/16.53/23.12/28.63/6.84) | (0.5345/0.3715/0.3348/0.5296/0.5397) | (15.37/0/0/15.78/23.49) |
| 15 |  | Cubic | -22340.41 | 44883.25 | (0/0/100/0/0) | (NaN/NaN/1/NaN/NaN) | (NaN/NaN/100/NaN/NaN) |

Abbreviations as in Supplemental Table 4.

**Supplemental Table 7: Multivariable linear regression analysis identifying independent cross-sectional associations between multiple risk factors and cIMT age 17 without multiple imputation for missing covariates of interest**

|  | Mean change in cIMT (mm) per 1-SD increase in risk factor | |
| --- | --- | --- |
|  | Beta (95% CI) | p value |
| Male Sex | 0.007 (0.004, 0.011) | < 0.001 |
| Height | 0.001 (-0.002, 0.003) | 0.675 |
| FFM | 0.011 (0.007, 0.014) | < 0.001 |
| FM | -0.004 (-0.006, -0.002) | < 0.001 |
| SBP | 0.005 (0.003, 0.007) | < 0.001 |
| CRP | -0.001 (-0.003, 0.000) | 0.097 |
| Physical Activity | -0.001 (-0.006, 0.003) | 0.537 |
| Dietary Intake | 0.000 (-0.003, 0.002) | 0.855 |

Values expressed as mean change in cIMT (mm) per z-score change in independent variable to allow direct comparison between different risk factors. Abbreviations: FFM, fat-free mass; FM, fat mass; SBP, systolic blood pressure; DBP, diastolic blood pressure; HDL-c, high-density lipoprotein cholesterol; CRP, C-reactive protein.

**Supplemental Table 8: Multivariable linear regression analysis identifying independent cross-sectional associations between multiple risk factors and cIMT and rIMT at age 21**

|  | Mean change in cIMT per 1-SD increase in risk factor (n=435) | | Mean change in rIMT per 1-SD increase in risk factor (n=175) | |
| --- | --- | --- | --- | --- |
|  | Effect estimate (95% CI) | p value | Effect estimate (95% CI) | p value |
| Male sex | 0.005 (-0.003, 0.013) | 0.221 | -0.002 (-0.013, 0.008) | 0.663 |
| Height | -0.002 (-0.010, 0.005) | 0.546 | -0.003 (-0.012, 0.006) | 0.546 |
| FFM | 0.011 (-0.001, 0.022) | 0.069 | 0.010 (-0.004, 0.023) | 0.156 |
| FM | -0.001 (-0.009, 0.006) | 0.702 | -0.001 (-0.010, 0.008) | 0.751 |
| SBP | 0.009 (0.003, 0.014) | 0.002 | 0.007 (0.001, 0.014) | 0.023 |
| CRP | 0.000 (-0.004, 0.005) | 0.869 | 0.001 (-0.005, 0.007) | 0.710 |

Abbreviations: FFM, fat-free mass; FM, fat mass; SBP, systolic blood pressure; DBP, diastolic blood pressure; HDL-c, high-density lipoprotein cholesterol; CRP, C-reactive protein.

**Supplemental Table 9: Longitudinal exposure to differing body compositions and systolic blood pressure throughout adolescence and IMT at age 17 without multiple imputation for missing covariates of interest**

|  | Model 1 | | Model 2 | |
| --- | --- | --- | --- | --- |
|  | Effect Estimate  (95% CI) | p-value | Effect Estimate  (95% CI) | p-value |
| Absolute FFM |  |  |  |  |
| Low | Reference | - | Reference | - |
| Middle | 0.002  (-0.002,0.006) | 0.351 | 0.003  (-0.003, 0.008) | 0.325 |
| High | 0.014  (0.011, 0.017) | < 0.001 | 0.007  (0.001, 0.014) | 0.021 |
| Absolute FM |  |  |  |  |
| Low | Reference | - | Reference | - |
| Middle | -0.001  (-0.004, 0.002) | 0.642 | -0.004  (0.000, -0.008) | 0.037 |
| High | -0.010  (-0.014, 0.006) | < 0.001 | -0.006  (-0.012, -0.001) | 0.013 |
| SBP |  |  |  |  |
| Low | Reference | - | Reference | - |
| Middle | 0.011  (0.007,0.015) | < 0.001 | 0.006  (0.001,0.011) | 0.012 |
| High | 0.008  (0.003,0.013) | 0.001 | 0.006  (0.000,0.012) | 0.046 |

FFM and FM were indexed to height^2^ to account for growth throughout adolescence. Model 1 – *Unadjusted;* Model 2 – *Model 1 + adjustments for sex, height, SBP, LDL-c, and CRP*

**Supplemental Figure 1: FFM trajectories across ages 9 to 17 years.** Trajectories have been derived from latent growth curve analysis, as described in detail in the Statistical Methods.

**
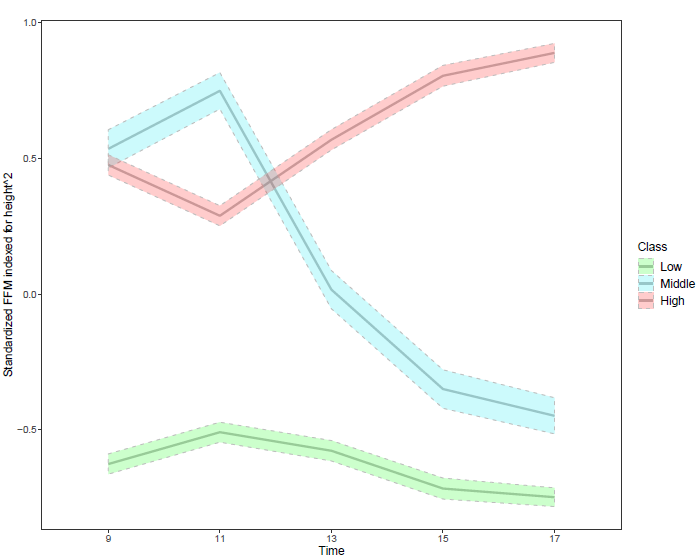
**

**Supplemental Figure 2: FM trajectories across ages 9 to 17 years.** Trajectories have been derived from latent growth curve analysis, as described in detail in the Statistical Methods.

**
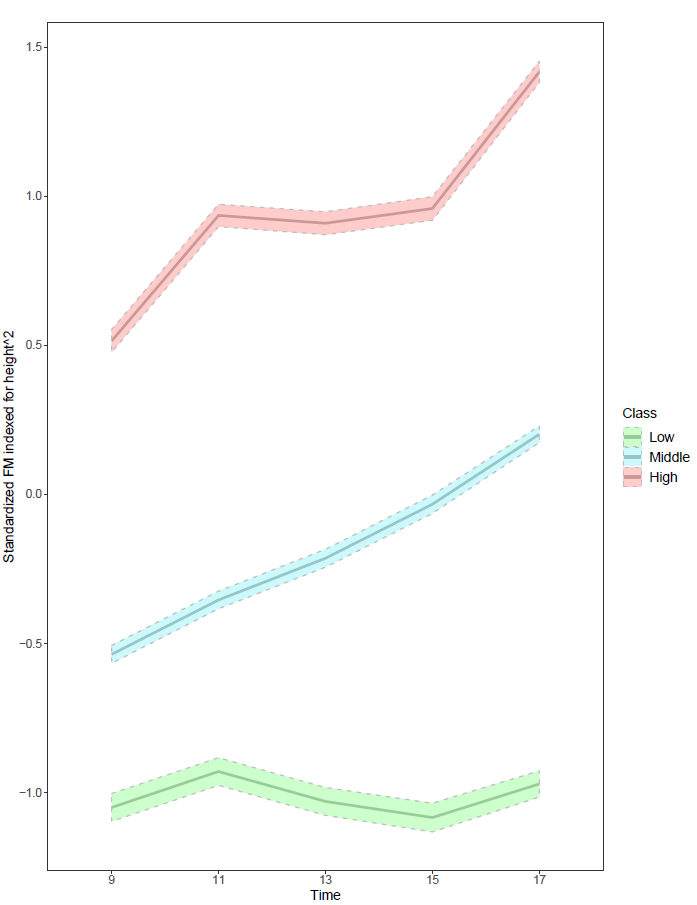
**

**Supplemental Figure 3: SBP trajectories across ages 9 to 17 years.** Trajectories have been derived **
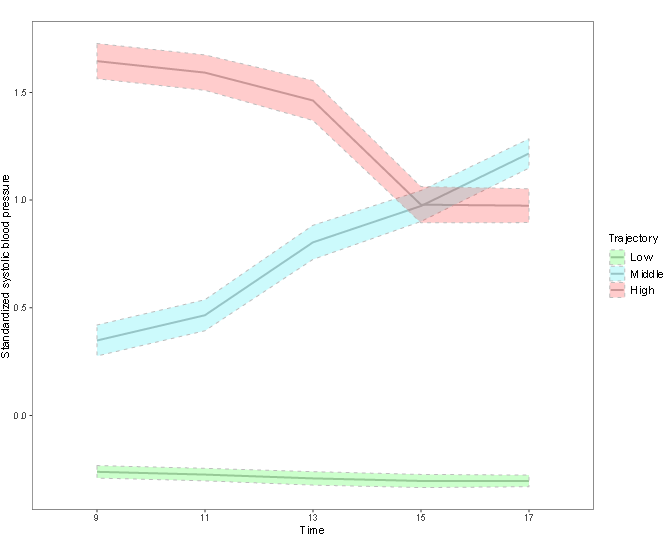
**from latent growth curve analysis, as described in detail in the Statistical Methods.

**Supplemental Figure 4: Carotid structure and stress at age 21**

**Supplemental Figure 5: Relationship between FFM and FM with different measures of IMT at in full cohort at ages 17 and 21 and in subsample of participants with UHFUS measures at 21.**





All variables adjusted for age, sex, height, and other tissue type.

**Supplemental Figure 6: Relationship between changes in body mass due to increasing FFM (blue) or FM (red) and radial media and intima thickness at 21**

All models adjusted for age, sex, and height
